# Arthroscopic lateral retinacular release improves patello-femoral and femoro-tibial kinematics in patients with isolated lateral retinacular tightness

**DOI:** 10.1007/s00167-021-06434-w

**Published:** 2021-01-26

**Authors:** Florian Pohlig, Ulrich Lenze, Florian Walter Lenze, Igor Lazic, Alexander Haug, Stefan Hinterwimmer, Heiko Graichen, Ruediger von Eisenhart-Rothe

**Affiliations:** 1 Department of Orthopaedic Surgery, Klinikum Rechts der Isar, Technical University Munich, Ismaninger-Strasse 22, 81675 Munich, Germany; 2 OrthoPlus Munich, Lenbachplatz 2a, 80333 Munich, Germany; 3 Hospital for Orthopaedic and Trauma Surgery, Lindenlohe 18, 92421 Schwandorf, Germany

**Keywords:** Patella, Lateral retinacular tightness, Kinematics, Lateral release, Arthroscopy, MRI, Patellar maltracking

## Abstract

**Purpose:**

Arthroscopic lateral retinacular release (LRR) has long been considered the gold standard for the treatment for anterior knee pain caused by lateral retinacular tightness (LRT). However, one-third of patients experience continuous pain postoperatively, which is thought to be related to persistent maltracking of the patella and altered femoro-tibial kinematics. Therefore, the aim of the present study was to simultaneously assess femoro-tibial and patello-femoral kinematics and identify the influence of arthroscopic LRR.

**Methods:**

Sixteen healthy volunteers and 12 patients with unilateral, isolated LRT were prospectively included. Open MRI scans with and without isometric quadriceps contraction were performed in 0°, 30° and 90° of knee flexion preoperatively and at 12 months after surgery. Patellar shift, tilt angle, patello-femoral contact area and magnitude of femoro-tibial rotation were calculated by digital image processing.

**Results:**

Postoperatively, patellar shift was significantly reduced at 90° of knee flexion compared to preoperative values. The postoperative patellar tilt angle was found to be significantly smaller at 30° of knee flexion compared to that preoperatively. Isometric muscle contractions did not considerably influence patellar shift or tilt in either group. The patello-femoral contact area increased after LRR over the full range of motion (ROM), with significant changes at 0° and 90°. Regarding femoro-tibial kinematics, significantly increased femoral internal rotation at 0° was observed in the patient group preoperatively, whereas the magnitude of rotation at 90° of knee flexion was comparable to that of healthy individuals. The pathologically increased femoral internal rotation at 30° without muscular activity could be significantly decreased by LRR. With isometric quadriceps contraction no considerable improvement of femoral internal rotation could be achieved by LRR at 30° of knee flexion.

**Conclusions:**

Patello-femoral and femoro-tibial joint kinematics could be improved, making LRR a viable surgical option in carefully selected patients with isolated LRT. However, pathologically increased femoral internal rotation during early knee flexion remained unaffected by LRR and thus potentially accounts for persistent pain.

**Level of evidence:**

II.

**Supplementary Information:**

The online version contains supplementary material available at 10.1007/s00167-021-06434-w.

## Introduction

Arthroscopic lateral retinacular release (LRR) has long been the treatment of choice for lateral patellar compression syndrome and patellar maltracking [30]. Nowadays, this diagnose complex is considerably differentiated and multiple factors have been identified to account for lateral patellar compression [4, 12]. Increase of patellar instability due to LRR and shortcoming of addressing all causative factors of the lateral patellar compression syndrome complex may lead to persistent maltracking [1, 12]. Accordingly, only isolated lateral retinacular tightness (LRT) is adequately addressed by LRR. However, one-third of these patients experience residual pain postoperatively indicating persistent patellar maltracking potentially influenced by pathological femoro-tibial kinematics [9].

Multiple analysis techniques for the identification of patellar kinematics have previously been applied [21, 23, 27]. Conventional radiography, however, has been proven to insufficiently assess the complex patellar motion patterns [12, 36]. 2D MRI and CT scans, on the other hand, significantly lack reproducibility regarding layer plane and orientation [4, 12]. Furthermore, 2D analysis techniques and fluoroscopy restrict the analysis to a single plane. Motion patterns perpendicular to the layer plane can rarely be assessed by these procedures, so it is not feasible to quantify 3D patellar kinematics or evaluate a potential relationship between femoro-tibial and patello-femoral motion patterns [7].

No in vivo studies have focused on the simultaneous analysis of patello-femoral and femoro-tibial kinematics in patients with LRT; yet, these data are critical for understanding the processes involved. Consequently, this knowledge may unveil if (1) patellar maltracking in patients with isolated LRT is caused by altered patello-femoral and femoro-tibial kinematics and if (2) pathological patellar tracking and femoro-tibial kinematics can be improved by arthroscopic LRR.

## Materials and methods

All patients and volunteers were provided with all relevant information before the beginning of the study, and written consent was obtained. The present study was approved by the institutional ethics committee of the Goethe University Frankfurt (IRB number 118/02).

Sixteen healthy volunteers (age 22–36 years; eight females, eight males) were recruited among the institutional staff and physiological values were determined. Healthy individuals had neither a history nor diagnosis of knee or hip pathology nor patello-femoral pain.

**Fig. 1:**
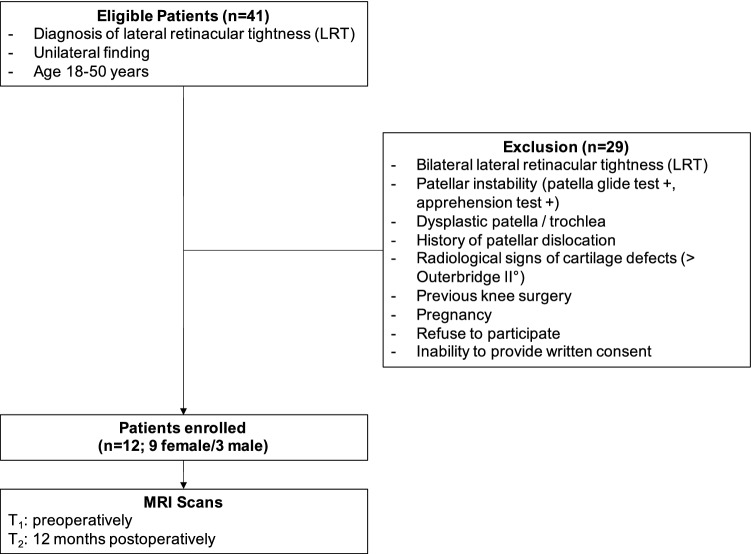
Flowchart of patient inclusion

Twelve patients (age 16–44 years; nine females, three males) with a diagnosis of unilateral, isolated LRT without patellar instability or a history of patellar dislocation were prospectively included in this study. All patients reported about pain during specific activities such as ascending and/or descending stairs, squatting, kneeling, jumping, long-sitting or running. Two independent senior orthopaedic surgeons diagnosed the cases on the basis of the patient’s medical history, clinical examination and conventional radiography as well as MRI findings. Specific in- and exclusion criteria are shown in Fig. 1. For the present study examinations and imaging scans were performed preoperatively and at 12 months after arthroscopic lateral release. In all cases, the diagnosis was confirmed intraoperatively.

### Surgical technique

All surgeries were performed under general anaesthesia. Standard arthroscopic portals were established and the joint was thoroughly evaluated. Chondral lesions were documented if present. Due to the exclusion of patients with cartilage defects greater than Outerbridge I, no cartilage reconstructions techniques had to be applied (Fig. 1). Subsequently, patellar tracking was evaluated arthroscopically, and then, LRR was performed using electrocautery. The release was carried out without affecting any muscle tissue. Conclusively, final patella tracking was verified.

Postoperatively, all patients were allowed to perform full weight-bearing as tolerated without any limitations in flexion. Physiotherapy was started immediately after the operation.

### Magnetic resonance imaging

Imaging scans were performed in an open MRI scanner (0.2T, MRT-open, Siemens, Erlangen) using an optimized T1-weighted 3D gradient echo sequence (TR = 16.1 ms; TE = 7.0 ms; Flipwinkel 30°) with a pixel size of 0.9 mm and a layer thickness of 1.9 mm. During the imaging scans, the patients were placed in lateral position and a positioning splint, ensuring the reproducibility of different flexion angles. Thus, the examined extremity was perfectly aligned with the longitudinal axis of the MRI scanner. Then, the patients were verified to be in the correct position with a localizer.

Initially, imaging of the affected knees was performed without muscular contractions in 0°, 30° and 90° of flexion. To investigate the influence of muscular activity during extension of the knee on patello-femoral kinematics, the procedure was repeated under isometric muscular activity. Therefore, an external weight was applied on the positioning splint with a torque of 10 Nm over the knee joint. Additionally, the patient had to keep contact with a specific area of the splint during the whole acquisition process to limit motion artefacts. Potential influence of muscular exhaustion was excluded by prior electromyography analysis.

### Digital image processing

The digital image processing technique has been previously published by our group [34]. In brief, three-dimensional semi-automated segmentation reconstruction of the bony structures (femur, tibia, patella) was performed. For additional calculations of the patello-femoral kinematics, a patella-based coordinate system was implemented, with the patellar centre of gravity as the origin.

The relative orientation and position of the patella in relation to the femoral bearing surface were defined automatically by femoral reference points. For the medial and lateral condyles, these points were configured by determining the shortest distance to the *y*–*z*-axis (frontal plane; *x* = 0) in the MRI data. For the depth of the femoral bearing trochlea, the point with the farthest distance from the *y*–*z*-axis (frontal plane; *x* = 0) was identified in accordance with the definition of the 1st femoral eigenvector by main axis transformation. Additionally, a local tibia-based coordinate system was applied according to the magnitude of tibial rotation. Finally, the tibia-based coordinate system and the femoral reference points were projected into the coordinate system of the patella. Thus, the quantification of the patellar alignment relative to the femur and tibia allowed an accurate characterization of the patellar kinematics by well-established 2D parameters.

The femoro-patellar contact area was calculated by separately segmenting the femoral and patellar cartilage. Second, all points in each layer with a distance of zero between the femoral and patellar cartilage surfaces were identified and collectively defined as the femoro-patellar contact area.

This MRI-based image processing technique has been extensively described in previous studies and a high reproducibility with a coefficient of variation (CV) of between 0.012 and 0.083 depending on the analysed parameter has been reported [10, 34].

Primary outcome parameters were improvement of patello-femoral maltracking, in particular patellar shift, tilt and contact area, as well as femoro-tibial rotation.

### Statistical analysis

All the data collected in this study were recorded and analysed using SPSS software (IBM, Armonk, NY). The normality of the data was assessed by the Shapiro–Wilk test. Pre- and postoperative results in the patient group were compared using the *t* test for paired samples (CI 95%). The patient and healthy control groups were compared using the *t* test for unpaired samples, with a 95% confidence interval. Regression analysis was performed to assess the impact of femoro-tibial rotation on the patella-femoral kinematics.

Sample sample-size power analysis of *β* = 0.20 and *α* = 0.05 was performed for a pilot study using mean differences for patellar tilt between the groups. Based on this analysis, a maximum of 11 participants per group was needed to power the study adequately.

## Results

### Patello-femoral kinematics

#### Patella shift

**Fig. 2:**
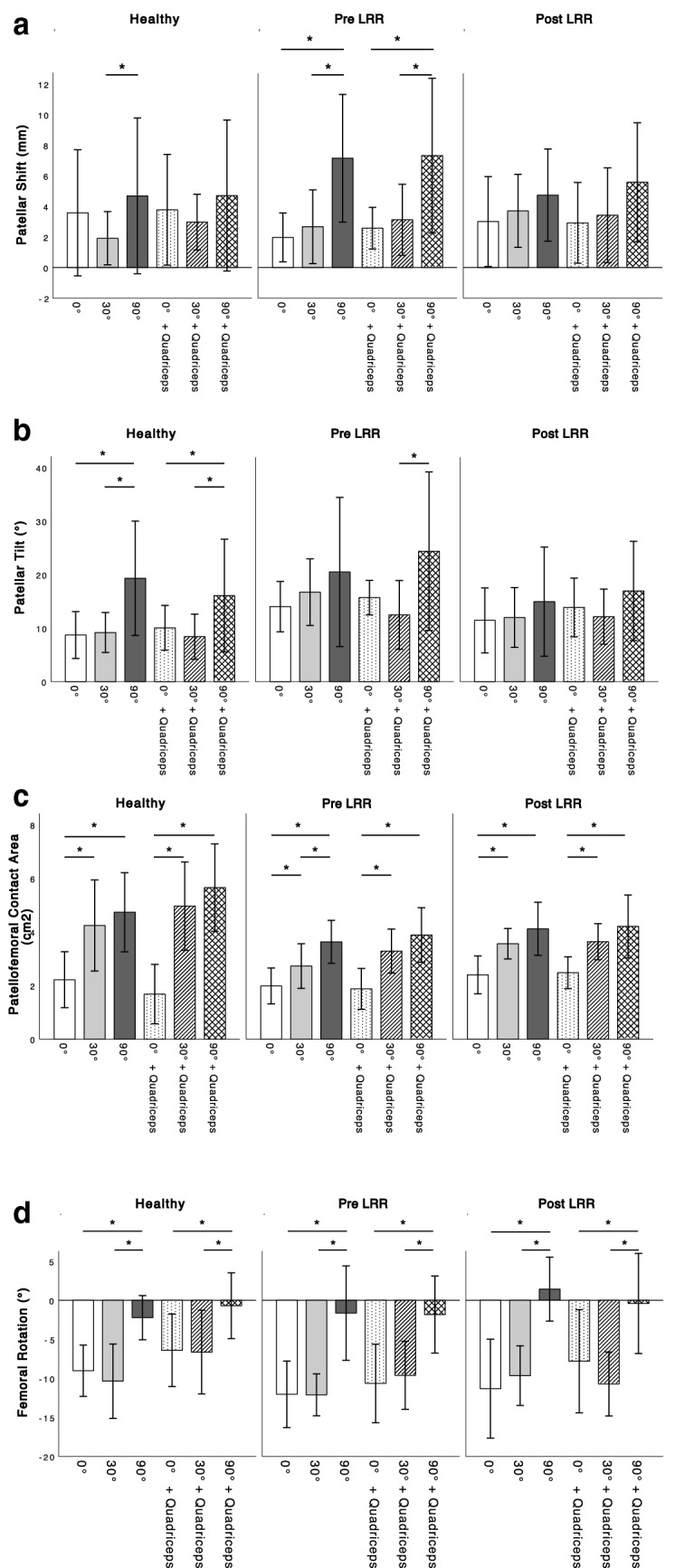
Patello-femoral and femoro-tibial kinematics in healthy knees and knees with LRT preoperatively and after arthroscopic LRR during knee flexion (0°–90°) with and without isometric muscle activity; **a** patellar shift; **b** patellar tilt; **c** patello-femoral contact area; **d** femoral rotation (negative values indicate femoral internal rotation based on a tibial coordinate system). *Statistical significance (*p* ≤ 0.05)

In the healthy knee joints a decrease in patellar shift was found from 0° to 30°, whereas a significant increase was identified at 90° of flexion (*p* = 0.046). Preoperatively, the knee joints of the patient group showed a continuous increase in patellar shift throughout knee flexion (Fig. 2a). Patellar shift slightly increased from pre- to postoperatively at 0° and 30° of flexion but significantly decreased at 90° of knee flexion (Table 1). The analysis of patellar shift with isometric muscle contraction revealed similar results.

**Table 1:** Patello-femoral and femoro-tibial kinematics in healthy knees and knees with LRT preoperatively and after arthroscopic LRR during knee flexion (0°—90°) with and without isometric muscle activity

Degree of flexion	0°	30°	90°	+ Quadriceps		
				0°	30°	90°
Healthy knees						
Patellar Shift (mm)	3.58 ± 4.12	1.92 ± 1.74	4.69 ± 5.09	3.78 ± 3.62	2.97 ± 1.82	4.71 ± 4.93
Tilt angle (°)	8.71 ± 4.39	9.18 ± 3.73	19.31 ± 10.69	10.05 ± 4.18	8.40 ± 4.21	16.09 ± 10.52
Femoropatellar contact area (cm^2^)	2.09 ± 0.89	4.31 ± 1.38	4.93 ± 1.26	1.63 ± 0.94	4.89 ± 1.54	5.78 ± 1.44
Femoral rotation (°)	− 9.02 ± 3.28	− 10.36 ± 4.77	− 2.22 ± 2.83	− 6.40 ± 4.65	− 6.64 ± 5.37	− 0.69 ± 4.21
Knees with LRT (pre-op)						
Patellar Shift (mm)	1.96 ± 1.53	2.74 ± 2.32	7.47 ± 4.16^+^	2.57 ± 1.36	3.12 ± 2.32	7.32 ± 5.06^+^
Tilt angle (°)	14.02 ± 4.71*	16.71 ± 6.23*	20.49 ± 13.95	15.69 ± 3.24*	12.47 ± 6.43*	24.37 ± 14.81
Femoropatellar contact area (cm^2^)	1.98 ± 0.67	2.73 ± 0.84	3.63 ± 0.80	1.88 ± 0.76	3.28 ± 0.82	3.89 ± 1.02
Femoral rotation (°)	− 12.04 ± 4.26*	− 12.12 ± 2.69	− 1.63 ± 6.05	− 10.65 ± 5.03*	− 9.62 ± 4.36	− 1.82 ± 4.94
Knees with LRT (post-op)						
Patellar Shift (mm)	2.90 ± 2.84	3.56 ± 2.34*	5.03 ± 3.07**	2.92 ± 2.64	3.42 ± 3.10	5.58 ± 3.89
Tilt angle (°)	11.44 ± 6.08	11.99 ± 5.58**	14.95 ± 10.21	13.87 ± 5.47*	12.14 ± 5.14*	16.95 ± 9.28
Femoropatellar contact area (cm^2^)	2.40 ± 0.71**	3.64 ± 0.68	4.12 ± 0.99**	2.48 ± 0.60	3.64 ± 0.67	4.21 ± 1.17
Femoral rotation (°)	− 11.32 ± 6.34	− 9.66 ± 3.82**	1.43 ± 4.10*	− 7.81 ± 6.61	− 10.73 ± 4.09*	− 0.41 ± 6.42

*Significant difference compared to healthy knees (*p* ≤ 0.05)

**Significant difference compared to preoperative values (*p* ≤ 0.05)

^+^Significant influence of femoral rotation (*p* ≤ 0.05)

#### Patella tilt

In healthy knee joints patella tilt angle slightly increased from 0° to 30° of flexion. A significant increase in patellar tilt was observed at 90° of flexion (Fig. 2b). The patient knee joints preoperatively showed a consistent, yet insignificant, increase throughout knee flexion (Fig. 2b). These values significantly differed from those in the control group at 0° and 30° (Table 1). The postoperative assessment revealed a decrease in patellar tilt at each measured degree of knee flexion compared to the preoperative values, with significant results at 30° of knee flexion (Table 1). Isometric quadriceps activity showed postoperatively significantly higher patellar tilt values at 0° and 30° of flexion in the patient group compared to the healthy control group (Table 1).

#### Patello-femoral contact area

A continuous increase of patello-femoral contact area throughout knee flexion could be observed in healthy individuals and the patient group (Fig. 2c). Preoperatively, smaller patello-femoral contact areas were found in the patient group compared to the control group. Following LRR significantly higher values were observed at 0° and 90° of flexion (Table 1). Isometric muscle contractions only had a minor effect on the patello-femoral contact area.

### Femoro-tibial kinematics

#### Femoral rotation

Healthy knees showed a marginal increase of femoral internal rotation from 0° to 30° and a significant decrease at 90°, corresponding to dorsal translation of the lateral condyle during larger degrees of flexion (Fig. 2d). Similar kinematics were identified in the patient group. After LRR, less femoral internal rotation was observed at any degree of flexion compared to preoperative values. Additionally, postoperative values of femoral rotation were found to be almost equal to those of the healthy control group in extension and at 30° flexion (Table 1). A significant reduction of femoral internal rotation was observed with isometric muscle contraction compared to without muscle contractions in healthy knee joints at 0° and 30° of flexion, whereas the patient group only showed a minor decrease in rotation preoperatively (Fig. 2d).

Regression analysis was performed to assess the influence of femoro-tibial rotation on patellar kinematics. In the healthy individuals, femoral rotation did not significantly influence patellar shift, patellar tilt or the contact area. Femoral rotation significantly influenced patellar shift preoperatively at 90° of flexion with and without isometric muscle contractions (Table 1). Postoperatively, no significant effects were observed.

## Discussion

The most important finding of the present study was that most aspects of patella-femoral and femoro-tibial joint kinematics could be improved by LRR in carefully selected patients with isolated unilateral LRT.

In the present study, an initial medialization of the patella from a more lateral position in full extension was followed by a subsequent increase in lateral patellar shift during knee flexion in the healthy control group. Similar results for healthy individuals were reported by previous studies [4, 20, 30]. The underlying mechanism is thought to be a medialization and engagement of the patella into the femoral trochlear groove, as the main static stabiliser, during early and mid-flexion. During higher degrees of flexion the passive stabilising function of the lateral retinaculum gains in importance and provokes a lateralisation of the patella [1]. Consequently, isolated LRT may only lead to an exuberant lateralisation of the patella during higher degrees of knee flexion. Confirming this hypothesis, the present study demonstrated an increased lateral patellar shift at 90° flexion in the patient group, which could be significantly reduced to values similar to those measured in the control group by LRR [24]. Furthermore, comparable values of lateral translation of the patella in full extension and at 30° knee flexion pre- and postoperatively underline the insufficiency of LRR to improve patellar shift at lower degrees of knee flexion.

Similar results were obtained for patella tilt angle with largest improvements from pre- to postoperatively at 90° of knee flexion. In contrast to other authors, however, relatively high patella tilt angles were identified in the present study [4, 14, 28]. On the other hand, in a systematic review, including only studies with CT- or MRI-based analysis techniques of patellar kinematics, Hochreiter et al. demonstrated a variation of the patella tilt angle from 0.7 ± 5.0° to 17.1 ± 4.3° in healthy individuals in full extension and 10° of flexion, respectively [12]. Besides differences in study cohorts, a lack of standardised imaging and measurement protocols was discussed as one of the main reasons for the highly variable results. In this context, several authors emphasized on the importance of 3D imaging techniques to consistently and precisely obtain landmarks, image plane location and orientation as several studies showed less accurate results for measurements on 2D images [11, 35]. Studies by Sebro et al., Mehl et al. and van Haver et al. are perfectly in line with our methods for healthy individuals [19, 31, 33]. Besides comparable measurement techniques Sebro et al. and van Haver et al. also presented good to excellent intraclass correlation coefficients (ICC) for patella tilt angle [31, 33].

Isometric muscular activity had a negligible effect on patellar shift and minor effect on patellar tilt angle in either group in the present study. Similar results were reported by Dupuy et al., who found a decrease in the lateral patella angle by 3% under load [2]. Moreover, a slight decrease in patellar tilt under muscular activity was also confirmed by Lorenz et al. [18]. Confirming these results, Lin et al. hypothesized that the relatively posterior patellar insertion of the vastus medialis oblique muscle (VMO) pulls the patella onto the femur during contraction leading to more rotational rather than translational displacement [17]. However, only flexion angles from 0° to 30° were investigated in their study. In higher degrees of knee flexion, the patella engages into the trochlea as the main static stabiliser. In individuals with a physiological anatomy of the femoral trochlea, the static stabilising function largely outweighs the impacts of the VMO [25]. This effect could be confirmed by Senavongse and Amis, as they identified a significantly larger loss of resistance to a lateral patella displacement by flattening of the lateral trochlea than by relaxation of the VMO [32]. Considering the diagnosis of isolated LRT without patellar instability or trochlea dysplasia in the present study, the aforementioned aspects could be confirmed.

Patello-femoral contact area significantly increased after arthroscopic lateral release in the present study. However, despite the improvement of patella tracking, complete restoration to physiological alignment did not occur. The present results are in line with those of a variety of other studies, even though comparism of the actual values to those in the other studies is limited due to the use of different examination techniques [22, 30, 34]. Isometric muscle contractions revealed slightly larger patellofemoral contact areas than did the absence of muscular activity, confirming trends reported in previously published studies, although Gold et al. reported considerably larger contact areas [6]. This inconsistency is probably due to Gold et al. including only male patients, who have clearly larger patello-femoral contact areas [6].

In healthy individuals, external femoral rotation during knee flexion corresponding to a posterior translation of the lateral condyle was found to occur from 30° to 90° of knee flexion in the present study. In line with these results, Hill et al. observed 15° of external femoral rotation from 60° to 110° of knee flexion whereas only 1° of external rotation occurred from 0° to 60° [8]. Similar data was presented in a recent meta-analysis by Galvin et al. [5]. Femoro-tibial rotation, in turn, has been proven to significantly influence patello-femoral kinematics [15, 16, 29]. In this context, Eckhoff et al. noted an increased external rotation of the tibia, corresponding to an internal rotation of the femur, in patients with anterior knee pain [3]. Confirming their results, larger angles of internal femoral rotation were preoperatively identified at 0° and 30° of knee flexion in the patient group compared to the control group. Besides other factors, these altered femoro-tibial kinematics may explain increased patella tilt angles and smaller patello-femoral contact areas in the patient group potentially accounting for characteristic anterior knee pain during specific activities like descending stairs, squatting, jumping, or running. Postoperatively, a significant reduction of the pathologically increased internal femoral rotation at 30° of knee flexion was observed. These results are consistent with those of previously published kinematic studies [8, 13, 26]. However, despite there being a significant reduction at 30° postoperatively in unloaded conditions, internal femoral rotation could not be completely restored to values measured in the healthy control group.

Isometric muscle contractions led to an increase in external femoral rotation in the patient and control groups. These results are in line with those of a meta-analysis regarding kinematic profiles in healthy knee joints in vivo under loaded conditions [5]. Similar results were found in an in vitro study by Li et al. with isolated quadriceps loading, specifically in extension, 30° and 60° [16]. Interestingly, in contrast to unloaded conditions, pathologically increased femoral internal rotation was not decreased by isometric quadriceps activity at 30° of knee flexion postoperatively, which is potentially attributed to a decreased lateral soft tissue tension due to the surgical release. This finding, in turn, may potentially explain persistent anterior knee pain in some patients despite improved patello-femoral kinematics.

However, several limitations of the present study have to be noted. First, LRT is not clearly defined but a diagnosis of exclusion. This can potentially lead to inclusion of patients with heterogenous patello-femoral pathologies which may influence the results. Second, patients with isolated LRT only represent a very small group within patients with anterior knee pain. Thus, the present results can only be very cautiously transferred to different patello-femoral conditions. Third, the present study was not performed as matched pair analysis. Considering the different gender ratio within the healthy individuals and the patient group, the comparison of absolute values has to be interpreted carefully. Fourth, in this study, the patients were examined only in a lying position without any axial loads, such as those incurred under physiological conditions, limiting further implications. Fifth, dynamic parameters could not be assessed by the techniques applied in this study due to insufficient quality of the data set acquired during dynamic MR techniques. Sixth, the acquisition time for a complete MR data set is approximately 4 min, and it is difficult for patients with patello-femoral pain to consistently hold isometric muscle contractions during the whole examination period. Moreover, the knee has to remain completely still for the duration of the examination to prevent artefacts.

## Conclusion

Patello-femoral and femoro-tibial joint kinematics could be improved, making LRR a viable surgical option in carefully selected patients with isolated LRT. However, pathologically increased femoral internal rotation during early knee flexion remained unaffected by LRR and thus potentially accounts for persistent pain.

## Supplementary Information

Below is the link to the electronic supplementary material.

Supplementary file1 (XLSX 26 KB)

## Abbreviations

CT: Computed tomography
LRR: Lateral retinacular release
LRT: Lateral retinacular tightness
MRI: Magnetic resonance imaging
ROM: Range of motion
VLO: Vastus lateralis oblique muscle
VMO: Vastus medialis oblique muscle
